# Genomic compatibility and inheritance of hexaploid‐derived Fusarium head blight resistance genes in durum wheat

**DOI:** 10.1002/tpg2.20183

**Published:** 2022-03-01

**Authors:** Xianwen Zhu, Jeffrey D. Boehm, Shaobin Zhong, Xiwen Cai

**Affiliations:** ^1^ Dep. of Plant Sciences North Dakota State Univ. Fargo ND 58108 USA; ^2^ USDA‐ARS Wheat, Sorghum and Forage Research Unit Lincoln NE 68583 USA; ^3^ Dep. of Plant Pathology North Dakota State Univ. Fargo ND 58108 USA; ^4^ current address: Institute of Advanced Agricultural Sciences Peking Univ. Weifang Shandong 261000 P. R. China

## Abstract

Hexaploid‐derived resistance genes exhibit complex inheritance and expression patterns in tetraploid backgrounds. This study aimed to characterize the inheritance patterns and genomic compatibilities of hexaploid‐derived Fusarium head blight (FHB) resistance genes in tetraploid durum wheat (*Triticum durum* Desf.). Evaluation of FHB resistance for F_1_ hybrids of hexaploid ‘Sumai 3’ crossed with tetraploid and hexaploid wheats indicated that Sumai 3‐derived FHB resistance genes exhibit a dominant phenotypic effect seen only in hexaploid hybrids. Alternately, the hexaploid‐derived FHB resistance genes from PI 277012 exhibited complete dominance in the crosses with both tetraploid and hexaploid wheat. FHB evaluation of the F_1_ hybrids of Sumai 3 and PI 277012 crossed with ‘Langdon’ (LDN)–‘Chinese Spring’ D‐genome substitution lines suggested that chromosomes 2B, 3B, 4B, 5B, 6B, 3A, 4A, 6A, and 7A contain genes that suppress expression of the Sumai 3‐derived FHB resistance, whereas chromosomes 4A, 6A, and 6B contain genes required for expression of PI 277012‐derived FHB resistance. A wide range of segregation for FHB severity (10–90%) was observed in the F_2_ generation of Sumai 3 crossed with durum cultivars LDN and ‘Divide’, but the distribution of F_3_ families derived from the most resistant F_2_ segregants was skewed towards susceptibility. Similar segregation trends were observed in the crosses of PI 277012 with other durum wheats, whereby FHB resistance became slightly diluted over successive generations. These results suggest tetraploid durum wheat contains the unique alleles at multiple gene loci on different chromosomes that positively and/or negatively regulate the expression of hexaploid‐derived FHB resistance genes, which complicate efforts to deploy these genes in durum breeding programs.

## Core Ideas

- Durum contains genes that enhance or suppress hexaploid‐derived FHB resistance.
- The hexaploid‐derived FHB resistance genes were inherited differently in durum.
- Genomic compatibility for FHB resistance was assessed by D genome substitutions.
- Sumai 3‐derived FHB resistance was suppressed by loci on nine durum chromosomes.
- Three durum chromosomes contain loci needed for PI 277012‐derived FHB resistance.

**Abbreviations**
CRD: completely randomized design
CS: ‘Chinese Spring’
FHB: Fusarium head blight
LDN: ‘Langdon’
PI: plant introduction
QTL: quantitative trait loci

## 1 INTRODUCTION

Fusarium head blight (FHB), also called scab, is one of the most devastating diseases in wheat (*Triticum aestivum* L.) worldwide. It is mainly caused by the fungus *Fusarium graminearum* Schwabe in North America. The major epidemic regions of FHB in the United States are in the mid‐western states comprising the Great Plains and in some eastern states (Stack, 1999). In the Great Plains, economic losses due to FHB from 1993 to 2001 were estimated at $2.5 billion dollars in wheat and barley (*Hordeum* L.) (Buerstmayr et al., 2020; Nganje et al., 2004). Globally, wheat yield losses due to FHB have been estimated as high as 21.5%, which was second only to leaf rust (Savary et al., 2019). Host plant resistance to FHB is considered the most efficient approach to reduce yield and quality losses compared to other management tactics, such as rotation and chemical control (McMullen et al., 1997; Rudd et al., 2001).

Fusarium head blight affects both hexaploid wheat (bread or common wheat) (*Triticum aestivum* L., 2n = 6x = 42, genome AABBDD) and tetraploid wheat (pasta or durum wheat) (*T. turgidum* L. ssp. *durum* Desf., 2n = 4x = 28, genome AABB). Despite successful identification of novel sources of FHB resistance in hexaploid wheat (He et al., 2013; Osman et al., 2015; Zhang et al., 2016; Ma et al., 2020), their subsequent deployment and usefulness in tetraploid wheat breeding programs have been scarce (Oliver et al., 2007). Therefore, there is an urgent need to identify and implement novel and effective FHB sources of resistance in tetraploid wheat.

To date, most of the commonly used sources of FHB resistance are derived from hexaploid wheat. ‘Sumai 3′, a Chinese hexaploid wheat cultivar, has been a widely used source of resistance to FHB in wheat breeding programs worldwide. Previous studies have implicated chromosomes 2B, 3B, 6B, and 7A harbored quantitative trait loci (QTL) and FHB resistance genes in Sumai 3 (Yao et al., 1997; Waldron et al., 1999; Zhou et al., 2002; Liu & Anderson, 2003). Among these genes, the major gene on 3B, designated *Qfhs.ndsu‐3BS* (*Fhb1*), has explained as much as 60.0% of the phenotypic variation for FHB resistance (Buerstmayr et al., 2002, 2009; Jayatilake et al., 2011). In addition, many other genes and QTLs have been associated in contributing both major and minor resistance to FHB; for example, between 2009–2019 alone, 64 different QTL mapping studies were conducted in wheat using linkage and association mapping procedures. QTL for FHB resistance have been identified on all wheat chromosomes, using differing inoculation and phenotyping procedures to assess Type I and Type II FHB resistance, FHB severity, FHB incidence, FHB index, deoxynivalenol accumulation and Fusarium‐damaged kernels (Bai et al., 1989; Van Ginkel et al., 1996; Buerstmayr et al., 2020; Ma et al., 2020; Zhu et al., 2020). Both Type II (resistance to the spread of the pathogen) and Type V (low accumulation of mycotoxins) resistance have been identified in Sumai 3 (Jayatilake et al., 2011).

**Core Ideas**

- Durum contains genes that enhance or suppress hexaploid‐derived FHB resistance.
- The hexaploid‐derived FHB resistance genes were inherited differently in durum.
- Genomic compatibility for FHB resistance was assessed by D genome substitutions.
- Sumai 3‐derived FHB resistance was suppressed by loci on nine durum chromosomes.
- Three durum chromosomes contain loci needed for PI 277012‐derived FHB resistance.

Preliminary studies seeking to clone the gene underlying resistance mechanisms of *Fhb1* were carried out, and a diagnostic molecular marker *UMN10* was developed by Liu et al. (2008). More recently, Su et al. (2019) and Li et al. (2019) reported that a mutation of a histidine‐rich calcium‐binding protein gene (named as *TaHRC*) at the *Fhb1* locus confers resistance against FHB. The introgression of FHB resistance from Sumai 3 has been successfully used to develop many hexaploid wheat germplasm and cultivars worldwide (He et al., 2001; Mergoum et al., 2006, 2008; Teresa et al., 2013; Waldron et al., 1999). In the 1980s, two hexaploid cultivars ‘Een 1′ and ‘Yangmai 4′ with moderate resistance to FHB were first developed from Sumai 3 and released in China (He et al., 2001). In the United States, the first FHB resistant cultivar, ‘Alsen’, derived from Sumai 3, was released in the late 1990s (Frohberg et al., 2006). After that, many more FHB‐resistant cultivars, including ‘Faller’, ‘Glenn’, and ‘Freyr’ were developed from Sumai 3 (Mergoum et al., 2006, 2008).

Recently, another hexaploid spring wheat accession with high level of FHB resistance ‘PI 277012′ was identified with a level of FHB resistance deemed equivalent to Sumai 3 (Chu et al., 2011). Molecular mapping research using doubled haploids derived from PI 277012 identified two FHB resistance QTL positioned on the short arm and long arm of chromosome 5A, respectively (Chu et al., 2011). Moreover, it appeared that the PI 277012‐derived FHB resistance genes could normally express when placed in a tetraploid wheat genetic background and hence could be very useful in tetraploid wheat breeding programs (Chu et al., 2011).

To identify novel FHB resistance sources in tetraploid wheat, extensive screening of wild relatives of tetraploid wheat has been carried out (Buerstmayr et al., 2012; Cai et al., 2005; Oliver et al., 2007, 2008; Ruan et al., 2012). For example, several FHB resistance QTL have been identified in wild emmer (*Triticum dicoccoides*) wheat accessions (Otto et al., 2002; Stack & Faris, 2006; Kumar et al., 2007). However, most of those identified QTL confer only moderate to low levels of resistance to FHB (Fakhfakh et al., 2011). Of these QTL, *Qfhs‐ndsu‐3AS* located on the short arm of chromosome 3A has been more extensively characterized than others (Chen et al., 2007; Otto et al., 2002; Zhu et al., 2015). The poor understanding of the chromosomal region where this QTL is positioned and the associated linkage drag of undesirable genes present in tetraploid wild relative introgressions has made it difficult to utilize these FHB resistance QTL directly in tetraploid wheat breeding programs.

One of the strategies to improve FHB resistance in tetraploid wheat is to transfer hexaploid‐derived FHB resistance genes to tetraploid wheat. However, progress using FHB resistance derived from Sumai 3 in tetraploid wheat breeding programs has been very limited. The possible mechanisms hindering the progress have been ascribed to (a) the difficulty in recovering the desired configuration of gene combinations for the successful introgression of Sumai 3 resistance (Liu & Anderson, 2003; Basnet et al., 2012); (b) complications arising from the absence of wheat D genome in tetraploid wheat, which possibly affects expression of FHB resistance genes upon introgression in a tetraploid genetic background (Rudd et al., 2001; Fakhfakh et al., 2011; Zhu et al., 2016); and, conversely, (c) suppressor genes found in tetraploid wheat's A or B genomes that may inhibit expression of Sumai 3‐derived FHB resistance (Rudd et al., 2001; Gilbert et al., 2002). For example, researchers were unable to utilize an introgression of tetraploid‐derived leaf and stem rust resistance genes when placed in a hexaploid genetic background due to the discovery of suppressor genes found on chromosomes 1D, 2D, 3D, and 4D that compromised the expression of the resistance (R) genes due to complementary gene action (Kerber & Green, 1980; Bai & Kbitt, 1992). Genomic incompatibilities provide yet another obstacle that breeders must overcome to successfully deploy newly discovered R genes via recombination, chromosomal translocations, or alien introgressions between tetraploid and hexaploid wheats. Hence, dissection of the genetic factors influencing expression of hexaploid‐derived FHB resistance genes in tetraploid wheat may facilitate improved utilization of hexaploid R genes in tetraploid breeding programs.

The objective of the present study is to investigate the effects and compatibility of durum's tetraploid genomic background on the expression and inheritance of hexaploid‐derived FHB resistance genes from Sumai 3 and PI 277012.

## 2 MATERIALS AND METHODS

### 2.1 Plant genetic materials and inheritance analysis

Four FHB‐susceptible tetraploid wheat cultivars—‘Langdon’ (LDN), ‘Grenora’, ‘Alkabo’, and ‘Divide’—were used as female parents in crosses made with Sumai 3 and PI 277012, two hexaploid wheat accessions highly resistant to FHB. The F_1_ hybrids along with their parents were evaluated for FHB severity in four greenhouse seasons (Fall 2011, Fall 2012, Spring 2013, and Spring 2014). Four hexaploid wheat accessions highly susceptible to FHB (‘2398′, ‘Choteau’, ‘AC Vista’, and ‘AC Lillian’) were also used as female parents to develop F_1_ hybrids with Sumai 3, which were evaluated for FHB resistance in two greenhouse seasons (Spring 2013 and Fall 2013).

In addition, a complete set of LDN‐‘Chinese Spring’ (CS) D‐genome substitution lines, developed by the USDA‐ARS Cereal Crops Research Unit, Fargo, ND, were crossed with Sumai 3 and PI 277012. In each substitution line, a pair of homologous A‐ or B‐genome chromosomes in LDN was substituted by a pair of its corresponding homoeologous D‐genome chromosomes from CS. The F_1_ hybrids of these crosses were evaluated for FHB resistance in either three (Spring 2013, Fall 2013, and Spring 2014) or two greenhouse seasons (Spring 2013 and Fall 2013).

F_1_ hybrids in all crosses were confirmed by visual inspection of seed morphology and molecular marker analysis. Some of the F_1_ hybrids were further verified by spike morphological comparisons. True F_1_ hybrids were employed for the evaluation of FHB resistance and advanced to the subsequent generations for inheritance studies.

To study inheritance patterns, evaluation of FHB resistance in segregating progeny was performed from the F_2_ to F_4_ generations for the crosses of LDN × Sumai 3, Divide × Sumai 3, and LDN × PI 277012. One spike of each plant was self‐pollinated to derive the next generation. The advanced F_5_ families were formed by the bulked seeds of resistant segregants identified in the previous generations.

### 2.2 Molecular marker analysis

Polymerase chain reaction (PCR) amplification was carried out in a 20‐μl mixture containing 40 ng genomic DNA, 0.5 μM each of forward and reverse primers, 1× PCR buffer, 1.5 mM MgCl_2_, 0.25 mM dNTP, and 0.25U of Taq DNA polymerase. Polymerase chain reaction was performed as the following protocol: 94 °C for 3 min; 45 cycles of 94 °C for 1 min, 55 °C for 1 min, and 72 °C for 1.5 min; then with a final 72 °C for 7 min. PCR products were separated on 8% polyacrylamide gel and visualized by ethidium bromide staining (Chen et al., 2007).

### 2.3 Experimental design and FHB evaluation

All plants were grown in 6‐in plastic pots with one plant per pot for each F_1_ hybrid, two plants per pot for parental controls, and each LDN‐CS D‐genome substitution line. Five to ten spikes in each pot were inoculated using the single floret inoculation method, described in detail below. The pots were randomly arranged on the greenhouse benches. Each pot was regarded as one replicate. The number of replicates for each entry in each experiment ranged from two to five depending on the successful germination of the F_1_ seeds. A completely randomized design (CRD) with unbalanced data was used for statistical analysis using SAS version 9.3 (SAS Institute, 2011). The data obtained in different greenhouse environments were pooled for combined analysis if the Bartlett's homogeneity test of error variance was not indicative of significant difference (*P* = .05).

The greenhouse temperature was maintained at approximately 16 and 18 °C at night and daytime, respectively, with a 16‐h photoperiod in the greenhouse before anthesis. During the inoculation period, the temperature was increased to approximately 25 °C. The single‐floret inoculation method was used to infect plants with the inoculum as described by Stack et al. (2002). For inoculation, a spore suspension at a concentration of 1 × 10^5^ conidiospores per ml was prepared as inoculum with a mixture of equal number of spores produced by four *F. graminearum* isolates (two 3ADON type isolates and two 15ADON type isolates), collected from North Dakota (Puri & Zhong, 2010), and ∼10 μl of the inoculum was injected into a central floret of each spike during anthesis. The inoculated spikes were covered with plastic bags, which were water‐misted on the inside to maintain a relatively high humidity to facilitate disease development for 72 h. At 21 d postinoculation, the percentage of diseased spikelets was recorded for each spike to evaluate Type II resistance and the mean percentage of FHB severity over all spikes in each pot was calculated and recorded as the value of one replicate.

## 3 RESULTS

### 3.1 Production of F_1_ hybrids

**FIGURE 1:**
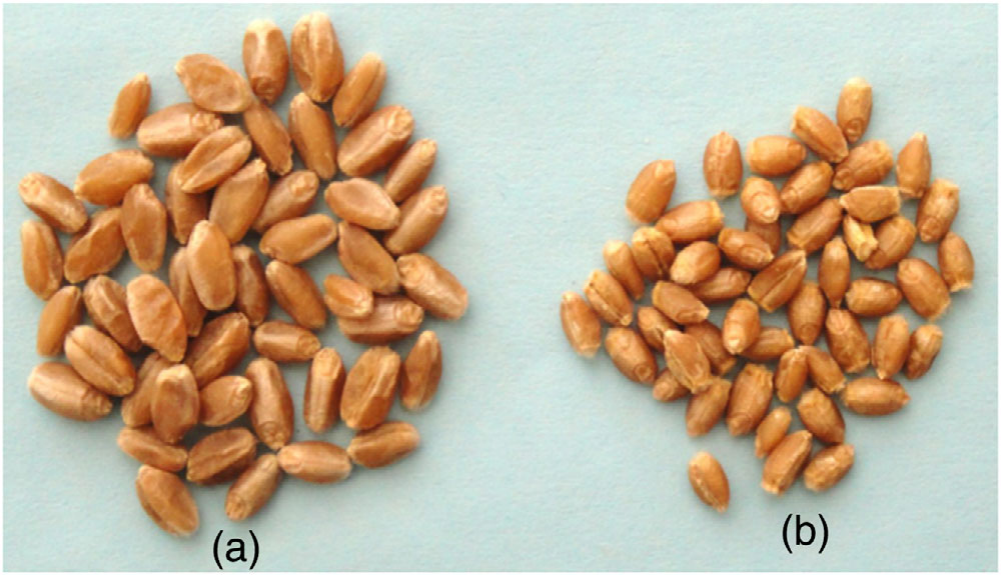
Illustration depicting significant seed size differences (*P* < .05) between (a) hexaploid cultivar ‘Choteau’ and (b) F_1_ hybrid of Choteau × Sumai 3

**FIGURE 2:**
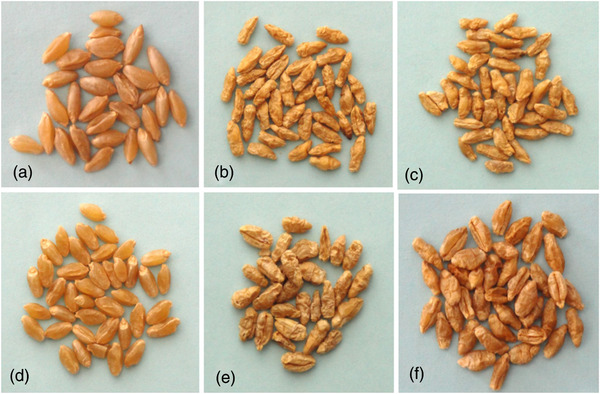
Illustration depicting seed morphological trait differences of tetraploid cultivars (a) ‘Langdon’ (LDN) and (d) ‘Divide’ compared to their F_1_ hybrids from the crosses of: (b) LDN × ‘Sumai 3’, (c) LDN × PI 277012, (e) ‘Divide’ × Sumai 3, and (f) Divide × PI 277012

**FIGURE 3:**
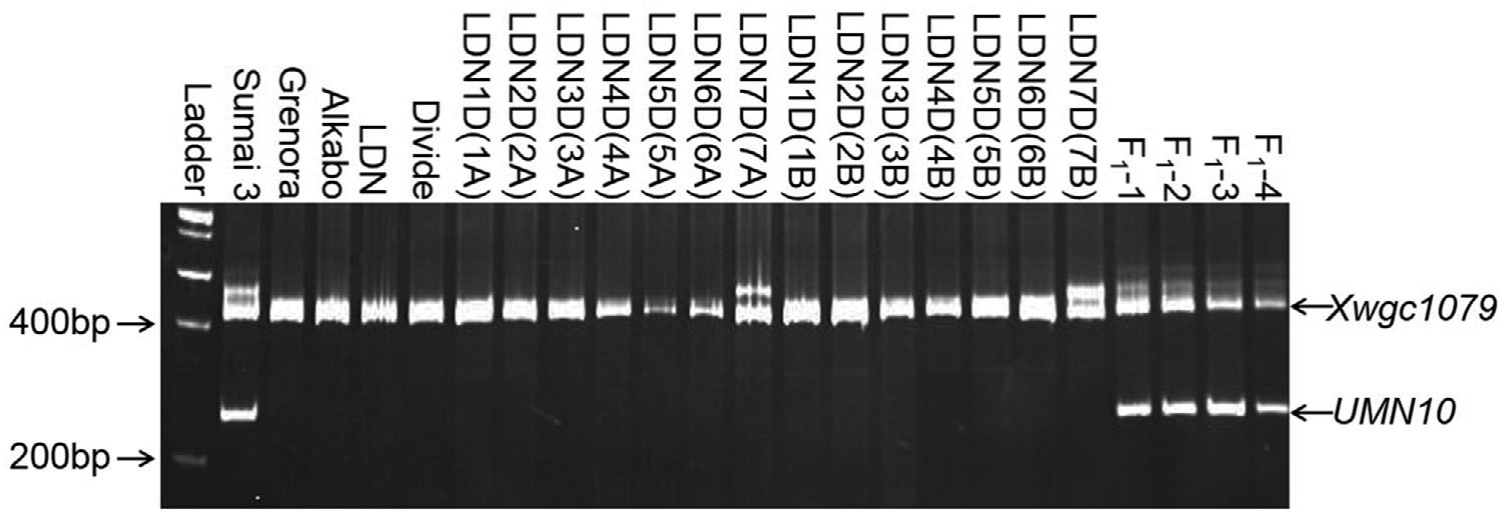
Polymerase chain reaction (PCR) amplification products from tetraploids and hexaploids at the molecular marker loci *UMN10* and *Xwgc1079* (internal control for PCR), respectively, for (from left to right): hexaploid cultivar ‘Sumai 3′, tetraploid cultivars ‘Grenora’, ‘Alkabo’, ‘Langdon’ (LDN) and ‘Divide’, the LDN‐Chinese Spring D‐genome substitution lines (*n* = 14), and tetraploid × hexaploid F_1_ hybrids from the crosses of LDN × Sumai 3 (F_1_‐1), Divide × Sumai 3 (F_1_‐2), LDN1D(1A) × Sumai 3 (F_1_‐3), and LDN2D(2A) × Sumai 3 (F_1_‐4)

**FIGURE 4:**
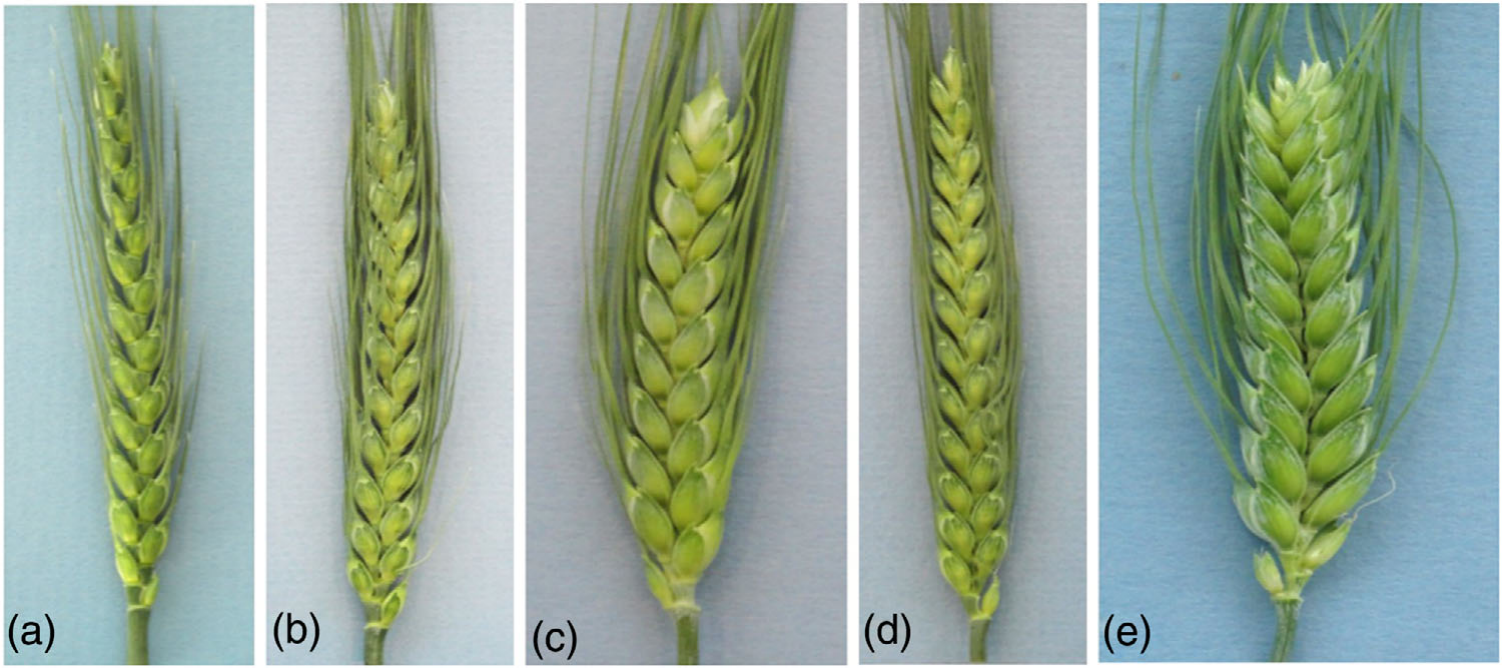
Illustration depicting spike morphological differences among the parents and their F_1_ hybrids. (a) PI 277012 (hexaploid); (b) F_1_ hybrid of ‘Divide’ × PI 277012 (tetraploid by hexaploid); (c) Divide (tetraploid); (d) F_1_ hybrid of ‘Langdon’ (LDN) × PI 277012 (tetraploid by hexaploid); (e) LDN (tetraploid)

Initially, the morphology of F_1_ hybrid seeds from all crosses was visually studied to validate that crossing was successful. True hybrid seeds derived from the crosses of hexaploid wheat lines 2398, Choteau, AC Vista, and AC Lillian by each of Sumai 3 and PI 277012 were significantly smaller than the normal self‐pollinated seeds of their female parents (Table 1; Figure 1). The true F_1_ seeds from the crosses of Sumai 3 and PI 277012 with tetraploid wheat lines LDN, Grenora, Alkabo, Divide, and the LDN‐CS D‐genome substitution lines were shriveled in comparison to the plump seeds of their female tetraploid parents (Figure 2). Ballester and de Vicente (1998) reported that molecular marker analysis is a more reliable method to verify presence of true F_1_ hybrids compared with other methods. Herein, molecular marker *UMN10*, which is commonly deployed by breeders for selection of *Fhb1* in Sumai 3, was used to validate presence of *Fhb1* at the seedling stage in each F_1_ hybrid derived from the crosses of Sumai 3 by tetraploid parents LDN, Grenora, Alkabo, Divide, and each LDN‐CS D‐genome substitution line. Each tetraploid wheat parent and each LDN‐CS D‐genome substitution line did not yield amplicons at the *UMN10* locus, whereas PCR amplification of products from each true F_1_ hybrid produced the same amplicon as Sumai 3 at the *UMN10* locus (Figure 3). The STS marker *Xwgc1079* (GATGGCCTAACAAATGATGT; TCCATCAAGCATACAGATGA) that constantly amplified a product (337 bp) in all genotypes involved in this experiment was used as an internal control for PCR (Figure 3). Lastly, spike morphology was used to confirm that crossing was successful for each F_1_ hybrid derived from the crosses of PI 277012 by tetraploid parents LDN, Grenora, Alkabo, Divide, and each LDN‐CS D‐genome substitution line. Visual observations confirmed presence of a speltoid (spear‐shaped) spike morphological phenotype for each true F_1_ hybrid derived from PI 277012, thereby confirming presence of the *q* gene on chromosome 5A from PI 277012 (Chu et al., 2011) (Figure 4).

**TABLE 1:** Mean seed morphological measurements of hexaploid wheat cultivars ‘2398’, ‘Choteau’, and ‘AC Vista’ and their F_1_ hybrids produced from crosses with hexaploid Fusarium head blight resistant cultivar ‘Sumai 3’

**Genotype**	**Sample size**	**Mean seed width**	**Mean seed length**
		mm	
2398	39	3.04 ± 0.27*a	5.97 ± 0.39a
F_1_ (2398 × Sumai 3)	50	2.76 ± 0.28b	4.34 ± 0.41b
Choteau	50	2.88 ± 0.31a	5.42 ± 0.43a
F_1_ (Choteau × Sumai 3)	50	2.39 ± 0.22b	4.14 ± 0.35b
AC Vista	27	2.80 ± 0.31a	6.50 ± 0.52a
F_1_ (AC Vista × Sumai 3)	34	2.43 ± 0.26b	4.8 ± 0.40b

* Mean ± standard deviation; Means followed by different letters for each F_1_ and its hexaploid parent are significantly different at α = 0.05.

### 3.2 FHB resistance in F_1_ hybrids derived from sumai 3

The F_1_ hybrids derived from the tetraploid (susceptible) by hexaploid (resistant) crosses of ‘LDN × Sumai 3′, ‘Divide × Sumai 3′, ‘Grenora × Sumai 3′ and ‘Alkabo × Sumai 3′ exhibited a resistance level similar to or lower than their tetraploid parents based on FHB severity ratings evaluated in one to three greenhouse environments (Table 2). An F_1_ hybrid derived from the cross Divide by Sumai 3 recorded an FHB severity rating of 38.7% that was significantly higher (*P* < .05) than that of Divide (28.0%). Statistical analyses indicated that the FHB severity rating for each of female parents LDN, Grenora, and Alkabo were not significantly different (*P* > 0.05) than that of their respective F_1_ hybrids derived from Sumai 3.

**TABLE 2:** Mean Fusarium head blight (FHB) severity of tetraploid cultivars ‘Langdon’ (LDN), ‘Divide’, ‘Grenora’, and ‘Alkabo’ and their F_1_ hybrids with ‘Sumai 3′

**Genotype**	**FHB severity**	**Greenhouse evaluation environment**
	%	
LDN	60.7b	Fall 2011, Spring 2013, Spring 2014
Sumai 3	18.4a^a^	
F_1_	59.1b	
Divide	28.0b	Fall 2011, Fall 2012
Sumai 3	17.1a	
F_1_	38.7c
Grenora	22.3ab	Fall 2011
Sumai 3	11.7a	
F_1_	31.4b	
Alkabo	29.7b	Fall 2011
Sumai 3	11.7a	
F_1_	32.9b	

^a^ Means followed by different letters are significantly different at α = 0.05 .

The F_1_ hybrids derived from highly susceptible hexaploid wheat cultivars 2398, Choteau, AC Lillian, and AC Vista by hexaploid resistant cultivar Sumai 3 exhibited a resistance level intermediate to that of their parents (Table 3). Mean FHB severity ratings of the four susceptible female parents ranged from 73.6% for AC Vista to 91.7% for 2398, whereas the mean FHB severity ratings of their F_1_ hybrids averaged ∼40%. In addition, the mean FHB severity rating for Sumai 3 was less than 15% in all greenhouse environments (Table 3). A combined data analysis across greenhouse environments was not performed with the spring wheat line 2398 due to failing Bartlett's Test for homogeneity of variances over the two greenhouse environments. 2398 exhibited a high mean FHB severity rating (91.7%) in one greenhouse environment, which was consistent with the report by Mergoum et al. (2008). However, this line had a significantly lower mean FHB severity rating (40.2%) in another greenhouse environment.

**TABLE 3:** Mean Fusarium head blight (FHB) severity of hexaploid wheat cultivars ‘2398’, ‘Choteau’, ‘AC Lillian’, and ‘AC Vista’ and their F_1_ hybrids with hexaploid FHB resistant cultivar ‘Sumai 3’

**Genotype**	**FHB severity**	**Greenhouse evaluation environment**
	%	
2398	91.7c	Fall 2013
Sumai 3	10.3a^a^	
F_1_	36.5b	
Choteau	77.0c	Spring 2013, Fall 2013
Sumai 3	14.3a	
F_1_	39.9b	
AC Lillian	74.3c	Spring 2013, Fall 2013
Sumai 3	14.3a	
F_1_	39.3b	
AC Vista	73.6c	Spring 2013, Fall 2013
Sumai 3	14.3a	
F_1_	38.1b	

^a^ Means followed by different letters within each set of three genotypes are significantly different at α = 0.05.

Most of the susceptible LDN‐CS D‐genome substitution lines and their F_1_ hybrids derived from resistant hexaploid wheat cultivar Sumai 3 were evaluated for FHB resistance in two or three greenhouse environments (Table 4). Three of the LDN–D‐genome chromosome substitution lines, including LDN2D(2A), LDN2D(2B), and LDN7D(7B), and their F_1_ hybrids derived from Sumai 3 were evaluated in only one greenhouse season due to seed shortage. The LDN‐CS D‐genome chromosome substitution lines of 3D(3A), 4D(4A), 6D(6A), 7D(7A), 2D(2B), 3D(3B), 4D(4B), 5D(5B), and 6D(6B) displayed significantly greater (*P* < .05) mean FHB severity than their F_1_ hybrids with Sumai 3. However, the F_1_ hybrids of LDN1D(1A), LDN1D(1B), LDN2D(2A), LDN5D(5A), and LDN7D(7B) with Sumai 3 showed a mean FHB severity level similar to or higher than their corresponding maternal substitution line (Table 4).

**TABLE 4:** Mean Fusarium head blight (FHB) severity of LDN‐CS D‐genome chromosome substitution lines, their F_1_ hybrids with hexaploid FHB resistant cultivar ‘Sumai 3’, and tetraploid cultivar LDN

**Genotype**	**FHB severity**	**Greenhouse evaluation environment**	**Genotype**	**FHB severity**	**Greenhouse evaluation environment**
**A genome**			**B genome**		
	%			%	
Sumai 3	15.4a^a^	Spring 2013, Fall 2013, Spring 2014	Sumai 3	15.4a	Spring 2013; Fall 2013; Spring 2014
F_1_	42.0b		F_1_	55.4b	
LDN1D(1A)	40.1b		LDN1D(1B)	52.9b	
LDN	58.6c		LDN	58.6b	
Sumai 3	21.8a	Spring 2014	Sumai 3	19.3a	Spring 2013
F_1_	87.6c		F_1_	52.2b	
LDN2D(2A)	91.2c		LDN2D(2B)	85.0c	
LDN	67.2b		LDN	52.4b	
Sumai 3	20.4a	Spring 2013, Spring 2014	Sumai 3	15.4a	Spring 2013; Fall 2013; Spring 2014
F_1_	57.6b		F_1_	40.0b	
LDN3D(3A)	69.0c		LDN3D(3B)	55.3c	
LDN	56.9b		LDN	58.6c	
Sumai 3	15.4a	Spring 2013, Fall 2013, Spring 2014	Sumai 3	15.4a	Spring 2013; Fall 2013; Spring 2014
F_1_	45.7b		F_1_	42.6b	
LDN4D(4A)	62.6c		LDN4D(4B)	55.5c	
LDN	58.6c		LDN	58.6c	
Sumai 3	15.4a	Spring 2013, Fall 2013, Spring 2014	Sumai 3	15.4a	Spring 2013; Fall 2013; Spring 2014
F_1_	52.8c		F_1_	38.0b	
LDN5D(5A)	30.2b		LDN5D(5B)	67.7c	
LDN	58.6c		LDN	58.6c	
Sumai 3	20.4a	Spring 2013, Spring 2014	Sumai 3	15.4a	Spring 2013; Fall 2013; Spring 2014
F_1_	65.9b		F_1_	62.2b	
LDN6D(6A)	84.4c		LDN6D(6B)	82.4c	
LDN	56.9b		LDN	58.6b	
Sumai 3	15.4a	Spring 2013, Fall 2013, Spring 2014	Sumai 3	21.8a	Spring 2014
F_1_	41.2b		F_1_	66.0b	
LDN7D(7A)	59.9c		LDN7D(7B)	60.0b	
LDN	58.6c		LDN	67.2b	

*Note*. LDN, ‘Langdon’.

^a^ Means followed by different letters within each set of four genotypes are significantly different at α = 0.05.

### 3.3 FHB Resistance in F_1_ hybrids derived from PI 277012

The F_1_ hybrids derived from the tetraploid (susceptible) × hexaploid (resistant) crosses of ‘LDN × PI 277012′, ‘Divide × PI 277012′, ‘Grenora × PI 277012′, and ‘Alkabo × PI 277012′ exhibited a resistance level not significantly different (*P* > .05) than paternal parent PI 277012 (Table 5). The mean FHB severity of all the F_1_ hybrids across populations was 14.3%, indicating complete dominance of the resistance genes in PI 277012 over the susceptible alleles in each maternal tetraploid parent. Comparison of the FHB mean severity ratings for the F_1_ hybrids derived from PI 277012 that were crossed with the complete set of LDN‐CS D‐genome substitution lines revealed that LDN4D(4A), LDN6D(6A), and LDN6D(6B) F_1_ hybrids had significantly higher (*P* < .05) mean FHB severity ratings than F_1_ hybrids of LDN × PI 277012, whereas the F_1_ hybrids derived from the remaining LDN‐CS D‐genome substitution lines exhibited similar levels of resistance as F_1_ hybrids derived from the cross of LDN × PI 277012 (Tables 5, 6).

**TABLE 5:** Mean Fusarium head blight (FHB) severity of tetraploid cultivars ‘LDN’, ‘Divide’, ‘Grenora’, and ‘Alkabo’ and their F_1_ hybrids with hexaploid FHB resistant line PI 277012

**Genotype**	**FHB severity**	**Greenhouse evaluation environment**
	%	
PI 277012	12.2a^a^	Fall 2011, Spring 2013
F_1_	15.1a	
LDN	57.9b	
PI 277012	12.2a	Fall 2011, Spring 2013
F_1_	15.3a	
Divide	41.0b	
PI 277012	9.4a	Fall 2011
F_1_	12.2a	
Grenora	22.3b	
PI 277012	9.4a	Fall 2011
F_1_	14.4a	
Alkabo	29.7b	

*Note*. LDN, ‘Langdon’.

^a^ Means followed by different letters within each set of three genotypes are significantly different at α = 0.05.

**TABLE 6:** Mean FHB severity of LDN‐CS D‐genome chromosome substitution lines, their F_1_ hybrids with hexaploid FHB resistant line PI 277012, and tetraploid cultivar LDN

**Genotype**	**FHB severity**	**Greenhouse evaluation environment**	**Genotype**	**FHB severity**	**Greenhouse evaluation environment**
**A genome**			**B genome**		
	%			%	
PI 277012	11.1a^a^	Spring 2013; Fall 2013	PI 277012	11.1a	Spring 2013; Fall 2013
F_1_	15.9a		F_1_	15.5a	
LDN1D(1A)	45.1b		LDN1D(1B)	57.7b	
LDN	56.4c		LDN	56.4b	
PI 277012	12.2a	Spring 2013	PI 277012	12.2a	Spring 2013
F_1_	16.9a		F_1_	22.0a
LDN2D(2A)	53.9b		LDN2D(2B)	85.0c	
LDN	52.4b		LDN	52.4b	
PI 277012	12.2a	Spring 2013	PI 277012	11.1a	Spring 2013; Fall 2013
F_1_	16.7a		F_1_	15.4a	
LDN3D(3A)	69.3c		LDN3D(3B)	54.6b	
LDN	52.4b		LDN	56.4b	
PI 277012	12.2a	Spring 2013	PI 277012	11.1a	Spring 2013; Fall 2013
F_1_	27.7b		F_1_	15.1a	
LDN4D(4A)	68.8d		LDN4D(4B)	62.8b	
LDN	52.4c		LDN	56.4b	
PI 277012	12.2a	Spring 2013	PI 277012	11.1a	Spring 2013; Fall 2013
F_1_	23.2a		F_1_	17.1a	
LDN5D(5A)	21.4a		LDN5D(5B)	62.7b	
LDN	52.4b		LDN	56.4b	
PI 277012	11.1a	Spring 2013; Fall 2013	PI 277012	11.1a	Spring 2013; Fall 2013
F_1_	19.9b		F_1_	24.8b	
LDN6D(6A)	96.0d		LDN6D(6B)	78.9d	
LDN	56.4c		LDN	56.4c	
PI 277012	10.4a	Fall 2013	PI 277012		Missing
F_1_	10.9a		F_1_		
LDN7D(7A)	50.4b		LDN7D(7B)		
LDN	62.3c		LDN		

*Note*. LDN, ‘Langdon’.

^a^ Means followed by different letters within each set of four genotypes are significantly different at α = 0.05.

### 3.4 Inheritance analysis

A total of 57 F_2_ plants from the cross of LDN × Sumai 3 were evaluated for FHB resistance by calculating the mean percentage of FHB severity ratings following the single‐floret spike inoculation method in the greenhouse (Stack et al., 2002). Of the 57 plants, two F_2_ plants with FHB severity ratings of 14.8 and 15.8%, respectively, were bulk harvested to form an F_3_ family consisting of 36 plants (Table 7). Of those 36 plants, two F_3_ plants with FHB severity ratings of 13.8 and 17.9%, respectively, were bulked to form an F_4_ family consisting of 38 plants. The same selection criteria were applied to the F_2_ population consisting of 37 plants from the cross of Divide × Sumai 3, which were evaluated for FHB resistance. In this case, four F_2_ plants were identified with FHB severity ratings from 7.4 to 16.3%, which were bulk harvested to form an F_3_ family consisting of 52 plants (Table 7). In the F_3_ generation, a single F_3_ plant with FHB severity rating of 12.9% yielded 22 seeds, which were used to establish an F_4_ family from the Divide × Sumai 3 cross. For the cross of LDN × PI 277012, 41 F_2_ plants were evaluated for FHB severity ratings. Of those 41 single plants, two F_2_ plants with FHB severity ratings of 6.8 and 8.4%, respectively, were selected and subsequently bulked to form an F_3_ family comprised of 59 plants (Table 7). In the F_3_ generation, two single plants were selected with FHB severity ratings of 12.9 and 15.0%, respectively, which were again bulk harvested to form an F_4_ family from the cross of LDN × PI 277012.

**TABLE 7:** Inheritance analysis of Fusarium head blight (FHB) resistance in three tetraploid × hexaploid populations derived from the crosses of susceptible tetraploid cultivars ‘Langdon’ (LDN) and ‘Divide’ by resistant hexaploid wheat cultivar ‘Sumai 3′ and resistant line PI 277012

**Population**	**Filial generation**	**Population size**	**Resistant plants**	**FHB severity of resistant plants**
				%
LDN × Sumai 3	F_2_	57	2	14.8, 15.8
	F_3_	36	2	13.8, 17.9
	F_4_	38		
Divide × Sumai 3	F_2_	37	4	7.4, 8.2, 14.5, 16.3
	F_3_	52	1	12.9
	F_4_	22		
LDN × PI 277012	F_2_	41	2	6.8, 8.4
	F_3_	59	2	12.9, 15.0
	F_4_	60		

*Note*. The populations were advanced using bulked seeds of resistant segregants identified in the previous generation. FHB severity ratings for resistant plants were calculated in a greenhouse experiment following the single‐floret spike inoculation method (Stack et al., 2002).

**FIGURE 5:**
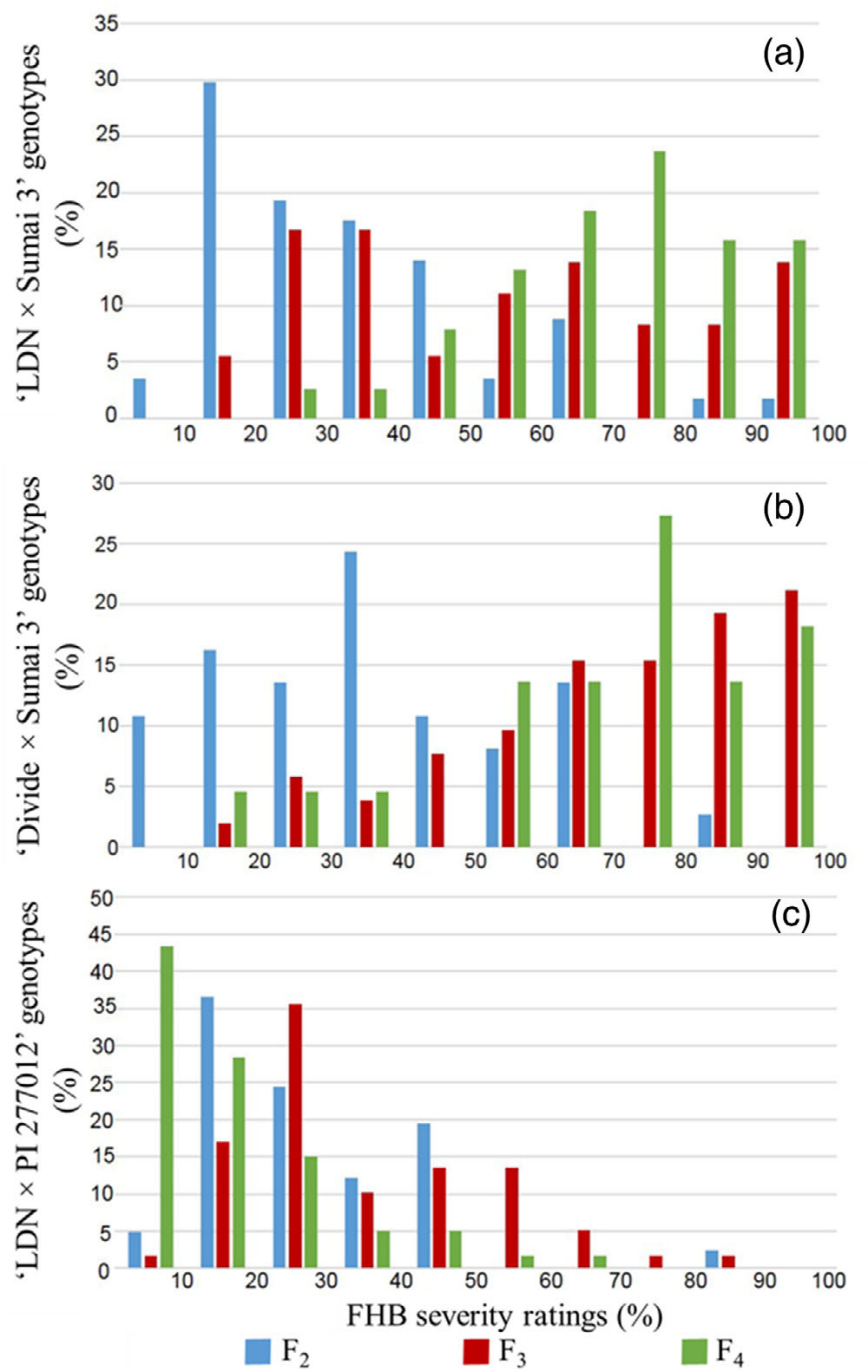
Distribution of Fusarium head blight (FHB) severity ratings observed in the F_2_, F_3_, and F_4_ generations in segregating susceptible tetraploid by resistant hexaploid populations derived from the crosses of (a) LDN × Sumai 3; (b) Divide × Sumai 3; (c) LDN × PI 277012. The populations were advanced using bulked seeds of resistant segregants identified in the previous generation

A wide variation in FHB severity was observed in the F_2_ population of the LDN × Sumai 3 cross. Approximately 53% of the plants exhibited FHB severity ratings of less than 30% and only 3.5% of the plants were identified with FHB severity ratings lower than 10% (Figure 5). All other segregants had FHB severity ratings that ranged from 30 to 90%. However, only 22.2% of the evaluated plants in the F_3_ generation and just 2.6% of the plants evaluated in the F_4_ generation were identified with an FHB severity rating of less than 30% (Figure 5). Individuals with an FHB severity rating of less than 20% were not observed in the F_4_ generation. In addition, over 50% and 80% individuals in the F_3_ and F_4_ families, respectively, were identified with FHB severity ratings higher than 50% (Figure 5). The F_3_ and F_4_ families in the cross of LDN × Sumai 3 had higher susceptibility compared with the F_2_ population.

Similar segregation patterns for FHB severity ratings were observed for families in the F_2_, F_3_, and F_4_ generations derived from the cross of Divide × Sumai 3 in comparison to that of LDN × Sumai 3. In the F_2_ population, approximately 25% of the individuals had FHB severity ratings of less than 20%. However, over 80% of the individuals exhibited an FHB severity rating greater than 50% in each of the F_3_ and F_4_ families (Figure 5).

For families derived from the cross of LDN × PI 277012, segregation patterns of FHB severity ratings were initially similar in the F_2_ generation to those patterns observed in the LDN × Sumai 3 and Divide × Sumai 3 crosses, respectively, as approximately 40% plants displayed an FHB severity rating of less than 20%. However, FHB severity ratings observed in the F_3_ and F_4_ generations clearly segregated differently than that of the LDN × Sumai 3 and Divide × Sumai 3 populations; for example, approximately 55% and 85% of plants in the F_3_ and F_4_ generations, respectively, recorded FHB severity ratings of less than 30%, which was a much higher frequency of resistant plants than that observed for the other two tetraploid by hexaploid populations (Figure 5).

## 4 DISCUSSION

Utilization of hexaploid‐derived FHB resistance in tetraploid wheat breeding programs has achieved limited success. It has been proposed that expression of hexaploid‐derived FHB resistance genes may be influenced by genetic factors present in tetraploid (Rudd et al., 2001; Fakhfakh et al., 2011). In this study, all F_1_ hybrids produced from the crosses of Sumai 3 with FHB susceptible hexaploid wheats, 2398, Choteau, AC Vista and AC Lillian, exhibited a level of FHB resistance intermediate to their respective parents. However, the F_1_ hybrids of Sumai 3 with tetraploid wheats LDN, Grenora, Alkabo, and Divide, exhibited levels of FHB resistance similar to or lower than that of their maternal tetraploid parent. Similar results were also reported in the F_1_ hybrids of other tetraploid wheat crossed with FHB‐resistant hexaploids including Sumai 3 (Gilbert et al., 2002). Conclusively, Sumai 3‐derived FHB resistance genes were normally expressed in the F_1_ hybrids produced from hexaploids, but not expressed in the F_1_ hybrids produced with the tetraploid cultivars as much as what were expressed in the hexaploid background. The mid‐parental FHB resistance level observed in the F_1_ hybrids of Sumai 3 with hexaploid wheat is consistent with polygenic inheritance in the hexaploid genetic background. It was proposed that suppression of Sumai 3‐derived resistance genes in tetraploid wheat was caused by suppressor genes in the tetraploid wheat genetic background (Gilbert et al., 2002). However, PI 277012‐derived FHB resistance genes are normally expressed in the F_1_ hybrids of each tetraploid wheat crossed with PI 277012 (Table 5). Apparently, FHB resistance genes in PI 277012 are completely dominant over the susceptible alleles in tetraploid wheat, which showed introgression efficiency of FHB resistance from various sources.

The F_1_ hybrids of Sumai 3 by the LDN‐CS D‐genome chromosomal substitution lines of LDN2D(2B), LDN3D(3A), LDN4D(4A), LDN4D(4B), LDN5D(5B), LDN6D(6A), LDN6D(6B), and LDN7D(7A) had significantly higher FHB resistance than their substitution line parents, which suggests LDN chromosomes 2B, 3B, 4B, 5B, 6B and 3A, 4A, 6A, and 7A, respectively, may contain genes that suppress expression of the Sumai 3‐derived FHB resistance genes. No significant increase of FHB resistance was observed in the F_1_ hybrids derived from LDN1D(1A), LDN2D(2A), LDN5D(5A), LDN1D(1B), and LDN7D(7B) by Sumai 3, indicating that LDN chromosomes 1A, 2A, 5A, 1B, and 7B may not influence expression of Sumai 3‐derived FHB resistance genes. The F_1_ hybrids derived from LDN4D(4A), LDN6D(6A), and LDN6D(6B) by PI 277012 exhibited an FHB resistance level lower than PI 277012, whereas the F_1_ hybrids of PI 277012 crossed with other substitution lines had a similar resistance level as PI 277012. These results suggest that LDN chromosomes 4A, 6A, and 6B likely contain genes required for expression of PI 277012‐derived FHB resistance. Moreover, these expression results clearly demonstrate that there are likely different mechanisms and/or different FHB resistance genes underlying the Sumai 3‐ and PI 277012‐derived FHB resistance. Zhuang et al. (2012) suggested that FHB resistance in Sumai 3 could be conferred by reducing the susceptibility rather than producing an active resistance reaction.

Wide variation for FHB severity ratings were observed in the F_2_ populations, F_3_ and F_4_ bulked families of the crosses of Sumai 3 by LDN and Divide. Furthermore, it was observed that the frequencies of plants with high levels of FHB resistance decreased from the F_2_ to the F_4_ generations. Alternately, a high frequency of plants with high levels of FHB resistance was retained over the F_2_ to F_4_ generations in the cross of PI 277012 by LDN. The difference in inheritance patterns between Sumai 3‐ and PI 277012‐ derived FHB resistance could be caused by several factors. Firstly, FHB resistance QTL have been identified on several chromosomes including 7A, 2B, 3B and 6B in Sumai 3 (Yao et al., 1997; Waldron et al., 1999; Zhou et al., 2002; Liu & Anderson, 2003). However, two FHB resistance QTL were mapped on the same chromosome 5A in PI 277012 (Chu et al., 2011). Thus, Sumai 3‐derived FHB resistance QTL recombine more frequently than those previously identified in PI 277012. Secondly, it was reported that introgression of a single FHB resistance gene from Sumai 3 could not provide an FHB resistance level comparable to Sumai 3 (Mergoum et al., 2006, 2008). However, one of the two QTL in PI 277012 mapped to chromosome 5A could provide a level of FHB resistance comparable with PI 277012 in other FHB susceptible wheat genetic backgrounds (http://www.uky.edu/Ag/Wheat/wheat_breeding/New%20Folder/Steve%20Xu.pdf). Lastly, herein we report that expression of Sumai 3‐derived hexaploid resistance genes was possibly suppressed by multiple genes on different tetraploid chromosomes. However, PI 277012‐derived FHB hexaploid resistance was normally expressed and the expression is possibly influenced by fewer genes in tetraploid.

## 5 CONCLUSION

The expression of hexaploid‐derived FHB resistance genes in Sumai 3 was suppressed by multiple genes on different chromosomes in a tetraploid genetic background, based on FHB severity ratings of segregating populations derived from tetraploid and hexaploid wheats and the complete set of LDN‐CS D‐genome chromosome substitution lines. Because of these genomic incompatibilities, it may be difficult to utilize Sumai 3‐derived FHB resistance genes for improvement of tetraploid wheat. However, complete dominance was observed for PI 277012‐derived resistance genes over the susceptible tetraploid alleles, indicating that introgression of these resistance genes would be less complicated. The differing inheritance patterns and expression of FHB resistance genes between Sumai 3 and PI 277012, observed herein, illustrate that proper selection of hexaploid FHB resistance donor sources could provide an opportunity to improve FHB resistance in tetraploid breeding programs.

## AUTHOR CONTRIBUTIONS

Xianwen Zhu: Data curation; Formal analysis; Methodology; Writing‐original draft. Jeffrey D. Boehm Jr.: Data curation; Writing‐review & editing. Shaobin Zhong: Investigation; Methodology; Resources. Xiwen D. Cai: Conceptualization; Data curation; Formal analysis; Funding acquisition; Investigation; Methodology; Project administration; Resources; Supervision; Validation; Visualization; Writing‐review & editing.

## CONFLICT OF INTEREST

All authors declare that they have no conflict of interest.
